# Supplementary material for: Antibiotics can be used to contain drug-resistant bacteria by maintaining sufficiently large sensitive populations
Source: PLoS Biol. 2020 May 15;18(5):e3000713. doi: 10.1371/journal.pbio.3000713 (PMC7266357; doi:10.1371/journal.pbio.3000713)
Supplement: S1 Text — (PDF) [file pbio.3000713.s001.pdf]

S1 Text: Details on parameter estimation

The resistant and sensitive strains were characterized using independent experiments that measured growth at different drug concentrations. Using a method detailed in [58], the CCD was configured to hold bacterial populations growing in different concentrations of drug at a constant density. The pump rate was fixed at 1 mL/min and then the pumps were turned on and off in order to maintain a constant OD. Growth rates were then determined by the average amount of time the pumps were on - pumps on more often indicates faster growth rates. If $r_S(D)$ and $r_R(D)$ are the growth rates of the sensitive and resistant strains at drug concentration D then:

$$r_S(D) = \frac{On_{pump}^{Avg}(D)}{V},$$

and

$$r_R(D) = \frac{On_{pump}^{Avg}(D)}{V},$$

where $On_{pump}^{Avg}(D)$ is the average amount of time the pumps are on for drug concentration D and V is the volume of the vial.

Using these estimated growth rates, we then fit a function (similar to a hill function) to characterize how each strain responds to different concentrations of drug. Specifically,

$$r_S(D) = \frac{r_S(0)}{\left(1 + \left(\frac{D}{h_S}\right)^{k_S}\right)} \left(1 - \frac{OD(D)}{C}\right) \tag{2}$$

and

$$r_R(D) = \frac{r_R(0)}{\left(1 + \left(\frac{D}{h_R}\right)^{k_R}\right)} \left(1 - \frac{OD(D)}{C}\right), \tag{3}$$

where $OD(D)$ is the the constant OD that the the population was held at when exposed to drug concentration D. Although different experiments were held at slightly different constant densities, $OD(D)$ was always very close to 0.02. In Equations (2) and (3), the term $\left(1 - \frac{OD(D)}{C}\right)$ is included to account for the fact that there may still be some competition at this low density.

In general, On_{pump}^{Avg} was higher at the beginning of an experiment and then decreased to a constant value, indicating that there is a time delay for drug to take effect. To account for this we used data between minutes 200 and 300 of the experiment to fit the above equations. By this time, the drug had reached its full effect and On_{pump}^{Avg} was essentially constant.

We also estimated the time delay associated with drug effect and included this in the main model (Model (1) from main text). The time delay for drug effect was estimated using the following equation:

$$r_S(D,t) = r_S(0) \left(1 - \frac{OD(D)}{C}\right) \frac{\left(1 + \left(\frac{D}{h_S}\right)^{k_S} \exp\left[-t/\tau_S\right]\right)}{\left(1 + \left(\frac{D}{h_S}\right)^{k_S}\right)},$$

and

$$r_R(D,t) = r_R(0) \left(1 - \frac{OD(D)}{C}\right) \frac{\left(1 + \left(\frac{D}{h_R}\right)^{k_R} \exp\left[-t/\tau_R\right]\right)}{\left(1 + \left(\frac{D}{h_R}\right)^{k_R}\right)}.$$

These equations were fit using all the data from an experiment that corresponded to a constant bacterial density. From these fits we obtained a time delay for the effect of drug for the sensitive (τ_S) and the resistant (τ_R) strain.
