# Supplementary material for: Antibiotics can be used to contain drug-resistant bacteria by maintaining sufficiently large sensitive populations
Source: PLoS Biol. 2020 May 15;18(5):e3000713. doi: 10.1371/journal.pbio.3000713 (PMC7266357; doi:10.1371/journal.pbio.3000713)
Supplement: S2 Text — (PDF) [file pbio.3000713.s002.pdf]

S2 Text: Details on mutation

Our experiments were seeded with a resistant OD of 0.02 (approximately 16000000 $\frac{\text{bacteria}}{\text{mL}}$). With this choice, the probability of mutation to resistance would have to be at least 0.056, and in practice much higher, before the cost of mutation would outweigh the benefit of competition. Alternatively, if the probability of mutation to resistance was 0.001 then the relative proportion of resistance at the beginning of the experiment would have to be less than 1 in 1000 (a situation that cannot be reliably created in our system) before there was even a possibility that maintaining a sensitive population would be detrimental. This means that within the operating regime of our experimental system and for the specific characteristics of the sensitive and resistant strains used, maintaining a sensitive population will never be detrimental. These calculations are described in more detail below.

Using a slightly modified version of our mathematical model of the bioreactor (Model 1 in main text), we can estimate what the probability of mutation would need to be before the presence of sensitive cells was detrimental. The dynamics of the resistant density in the mixed vial is given by:

$$\dot{R} = \frac{r_R}{1+\left(\frac{D(t-\tau_R)}{h_R}\right)^{k_R}}\left(1-\frac{S+R}{C}\right)R - \frac{F_D\chi_D+F_N}{V}R. \tag{4}$$

If the probability of mutation to resistance is ϵ, then adding mutational input to Equation (4) results in:

$$\begin{aligned}\dot{R} &= \frac{r_R}{1+\left(\frac{D(t-\tau_R)}{h_R}\right)^{k_R}}\left(1-\frac{S+R}{C}\right)R + \underbrace{\frac{\epsilon r_S}{1+\left(\frac{D(t-\tau_S)}{h_S}\right)^{k_S}}\left(1-\frac{S+R}{C}\right)S}_{\text{mutational input}} \\ &\quad -\frac{F_D\chi_D+F_N}{V}R. \end{aligned}\tag{5}$$

Equation (5) can be rewritten to isolate the effect that sensitives have on the resistant population:

$$\begin{aligned}\dot{R} &= \frac{r_R}{1+\left(\frac{D(t-\tau_R)}{h_R}\right)^{k_R}}\left(1-\frac{R}{C}\right)R - \frac{F_D\chi_D+F_N}{V}R \\ &\quad \underbrace{+\underbrace{\frac{\epsilon r_S}{1+\left(\frac{D(t-\tau_S)}{h_S}\right)^{k_S}}\left(1-\frac{S+R}{C}\right)S}_{\text{mutational input}} - \underbrace{\frac{r_R}{1+\left(\frac{D(t-\tau_R)}{h_R}\right)^{k_R}}\frac{S}{C}R}_{\text{competitive suppression}}}_{\text{effect of sensitive population on resistant population}}. \end{aligned}\tag{6}$$

Therefore, the benefit of competitive suppression will exceed the cost of mutation whenever

$$\frac{r_R}{1+\left(\frac{D(t-\tau_R)}{h_R}\right)^{k_R}}\frac{S}{C}R > \frac{\epsilon r_S}{1+\left(\frac{D(t-\tau_S)}{h_S}\right)^{k_S}}\left(1-\frac{S+R}{C}\right)S.$$

We can use the above relation to obtain a lower bound for how much mutation there must be before there is any risk of the sensitive population being detrimental. Specifically,

$$\epsilon_{min} = \frac{r_R}{r_S}\frac{1}{1+\left(\frac{D_{max}}{h_R}\right)^{k_R}}\frac{R(0)}{C-P_{max}} < \frac{r_R}{r_S}\frac{1+\left(\frac{D(t-\tau_S)}{h_S}\right)^{k_S}}{1+\left(\frac{D(t-\tau_R)}{h_R}\right)^{k_R}}\frac{R}{C-P_{max}},$$

where $D_{max} = 125 \frac{\text{ng}}{\text{mL}}$ is the maximum drug concentration allowed in the vials and $R(0)$ is the starting resistant density. Using the parameter values for our model this gives $\epsilon_{min} = 0.056$ when $P_{max} = 0.1$ and $\epsilon_{min} = 0.094$ when $P_{max} = 0.2$.

Note that ϵ_{min} is a lower bound for how high the probability of mutation must be before sensitive cells are detrimental. In practice, ϵ could be higher than this and the net effect of the sensitive population (over the entire course of the infection) could still be to delay resistance emergence.

Alternatively, if we know the probability of mutation to resistance then we can compute an upper bound for the starting resistant density:

$$\begin{aligned} R_{max} &= \epsilon\left(C - P_{max}\right)(1.42) = \epsilon\left(C - P_{max}\right)\frac{r_S}{r_R}\left(1 + \left(\frac{D_{max}}{h_R}\right)^{k_R}\right), \\ &> \epsilon\left(C - P_{max}\right)\frac{r_S}{r_R}\left(\frac{1 + \left(\frac{(t-\tau_R)}{h_R}\right)^{k_R}}{1 + \left(\frac{D(t-\tau_S)}{h_S}\right)^{k_S}}\right). \end{aligned} \tag{7}$$

Therefore, if the probability of mutation to resistance is 0.001 then the starting OD for the resistant strain would have to be less than 0.0002 (for $P_{max} = 0.2$) and less than 0.00035 (for $P_{max} = 0.1$) before there is any risk of mutational costs exceeding competitive benefits.
