# Supplementary material for: Antibiotics can be used to contain drug-resistant bacteria by maintaining sufficiently large sensitive populations
Source: PLoS Biol. 2020 May 15;18(5):e3000713. doi: 10.1371/journal.pbio.3000713 (PMC7266357; doi:10.1371/journal.pbio.3000713)
Supplement: S3 Text — (PDF) [file pbio.3000713.s003.pdf]

S3 Text: Drug free control populations

In addition to the experimental populations grown in each bioreactor experiment (described in Fig 3A), we also measured growth in 3 control populations grown in neighboring bioreactor vials. One vial contained the resistant strain (starting at the same initial density as in the resistant-only experimental population) but experienced no influx or outflow of media and received no drug. These unperturbed growth curves are shown in S2 Fig. A second control vial contained the resistant strain (again starting at the same initial density as in the resistant-only experimental population) and received inflow and outflow identical to that received in the experimental populations. However, the drug inflow solution was replaced by drug-free media. A third vial was identical to the second, but also contained sensitive cells and therefore served as a drug-free matched control of the mixed experimental population. The latter two controls eclipse the density threshold in each experiment (S3 Fig), indicating that the adaptive therapies leading to containment inhibit growth via drug rather than via rapid influx and outflow.
