# Supplementary figures and images for: Antibiotics can be used to contain drug-resistant bacteria by maintaining sufficiently large sensitive populations
Source: PLoS Biol. 2020 May 15;18(5):e3000713. doi: 10.1371/journal.pbio.3000713 (PMC7266357; doi:10.1371/journal.pbio.3000713)

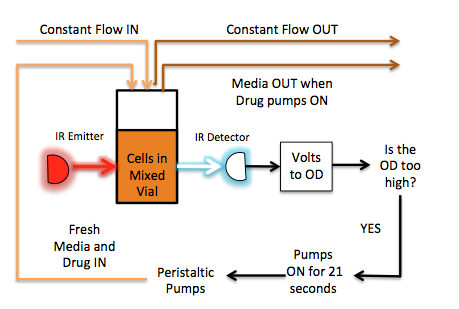

Supplement: S1 Fig — Constant-volume bacterial cultures (17 mL) are grown in glass vials with customized Teflon tops that allow inflow and outflow of fluid via silicone tubing. Flow is managed by a series of computer-controlled peristaltic pumps that are connected to media and drug reservoirs. Cell density is monitored by light scattering using infrared LED/detector pairs on the side of each vial holder. Voltage readings are converted to OD using a calibration curve based on separate readings with a tabletop OD reader. Up to nine cultures can be grown simultaneously using a series of multiposition magnetic stirrers. The entire system is controlled by custom Matlab software. Flow chart (above) depicts adaptive drug therapy (lower branches) intended to maintain constant OD by adding drug in response to changes in cell density. LED, light-emitting diode; OD, optical density. (PNG) [file pbio.3000713.s004.png]

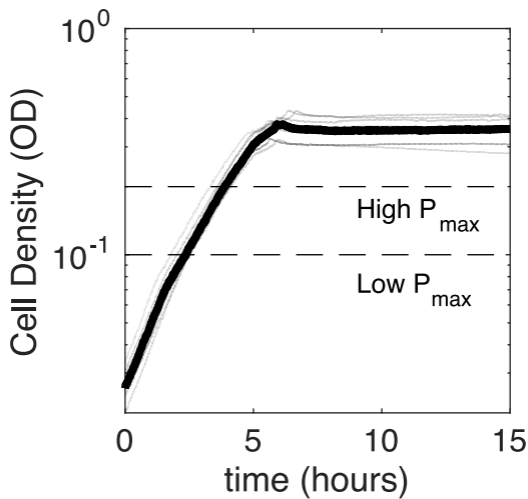

Supplement: S2 Fig — Cell density (OD) over time for REL607-derived resistant strains in bioreactors without influx or outflow of media. Transparent black lines correspond to growth curves performed in parallel with each bioreactor experiment. Thick black curve is the median over replicates. Dashed lines indicate threshold densities used in experiments (Pmax = 0.2 and Pmax = 0.1). Data are deposited in the Dryad repository: https://doi.org/10.5061/dryad.s4mw6m943 [62]. OD, optical density; Pmax, acceptable burden. (PDF) [file pbio.3000713.s005.pdf]

A

High Density Regime

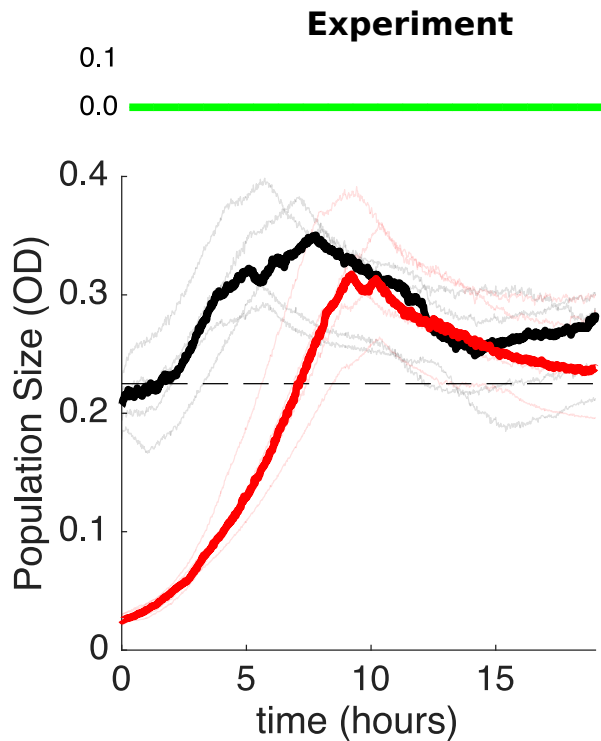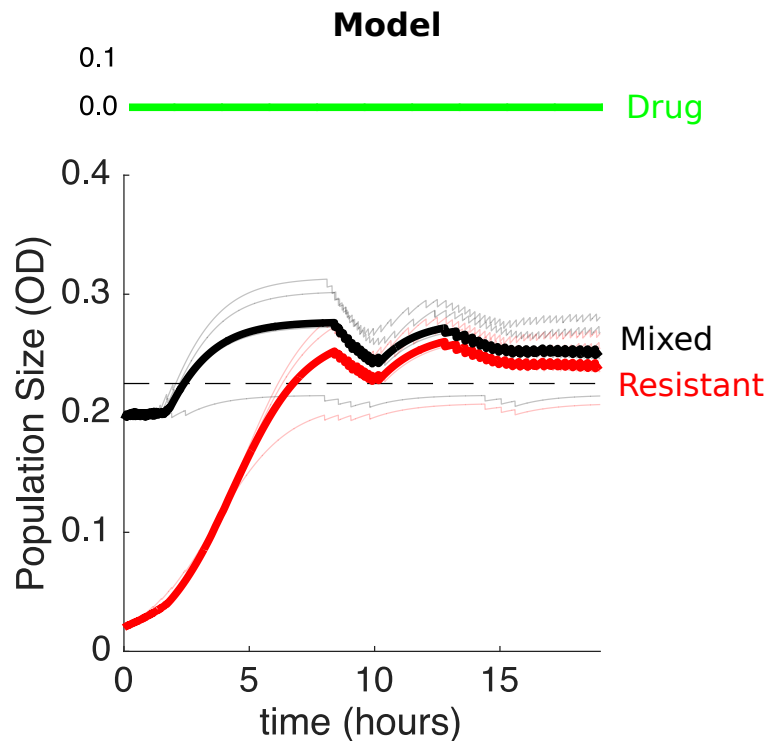

B

Low Density Regime

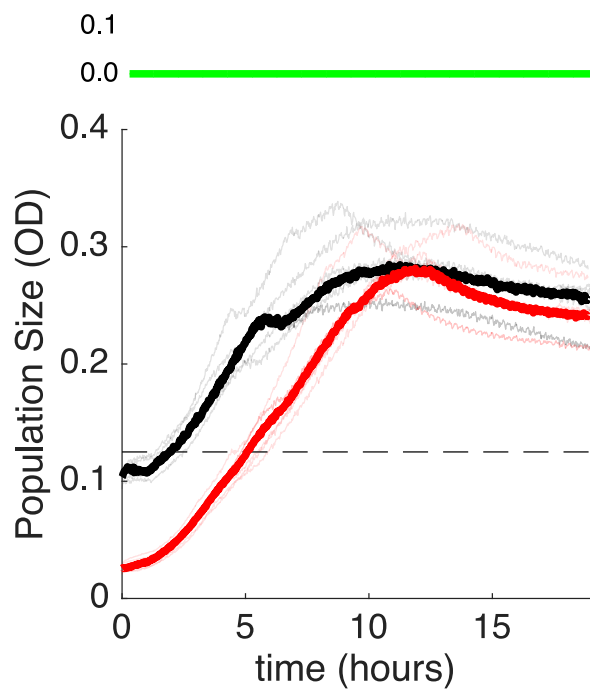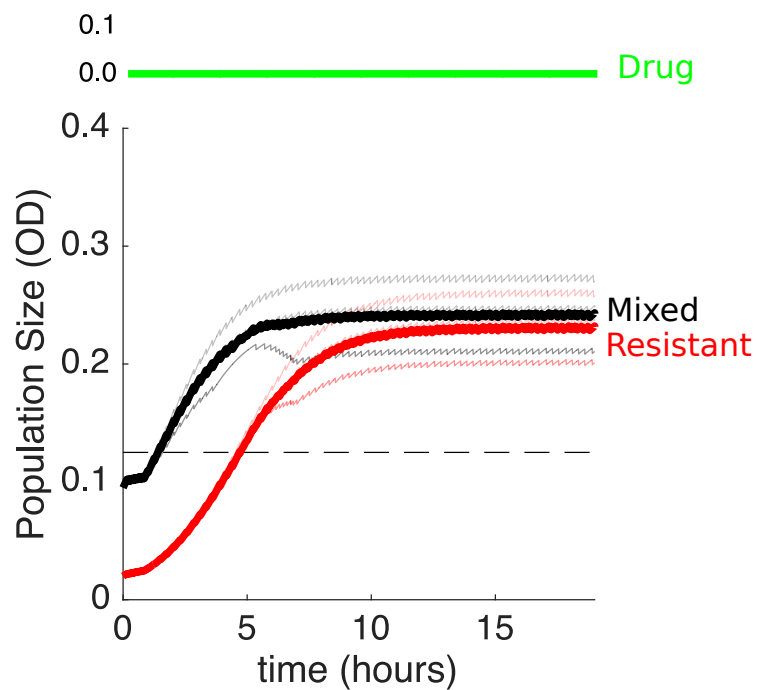

Supplement: S3 Fig — Conditions are identical to those in Fig 3B and 3C except that all populations receive drug-free media rather than drug solution media as part of the adaptive dosing protocol. Data are deposited in the Dryad repository: https://doi.org/10.5061/dryad.s4mw6m943 [62]. (PDF) [file pbio.3000713.s006.pdf]

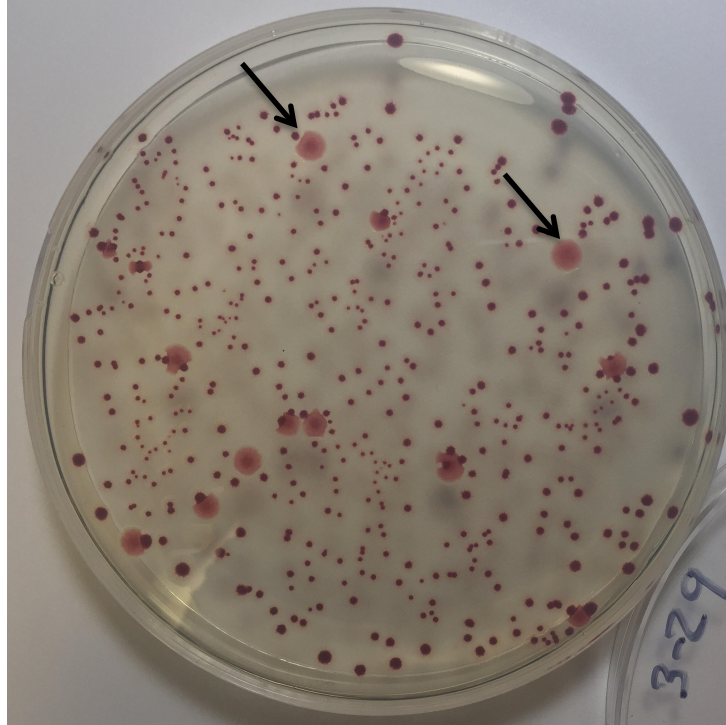

Mixed

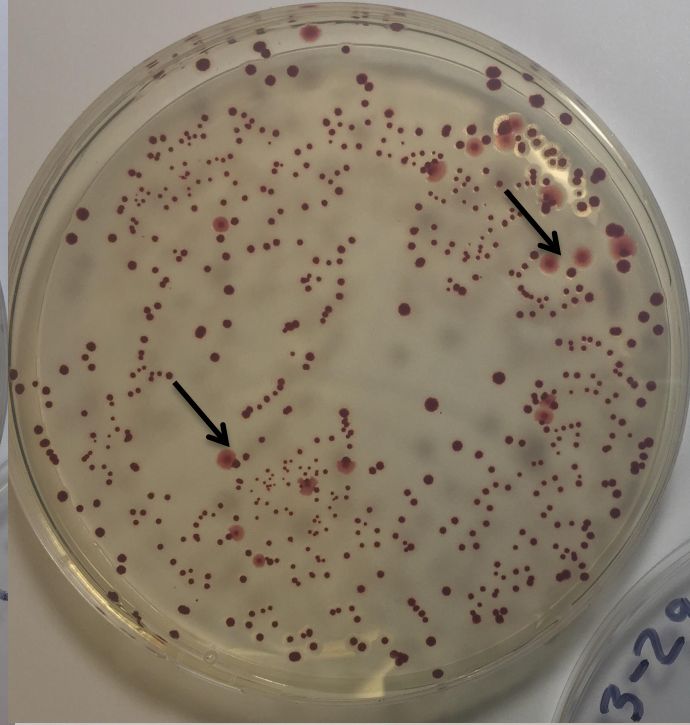

Mixed

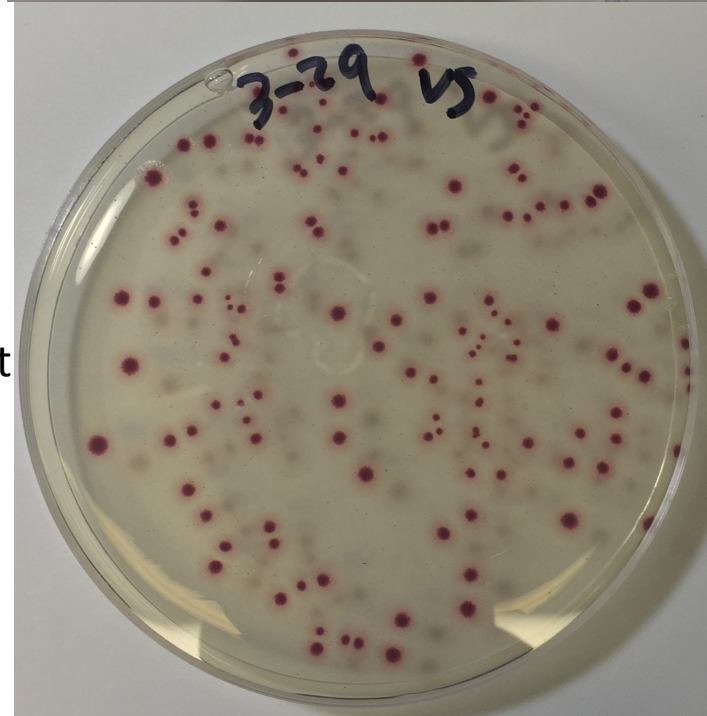

Resistant  
Only

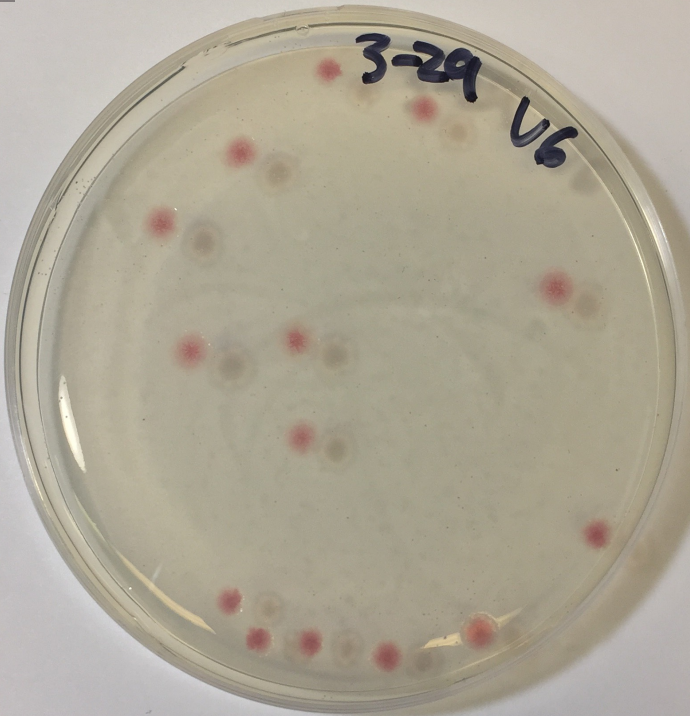

Sensitive  
Only

Supplement: S4 Fig — The REL606-derived resistant strain appears red, and the sensitive REL607 strain appears pink when grown on TA plates. Upper panels: samples from two mixed vials taken at the end of a high-density escape time experiment (as in Fig 3B). Arrows indicate sensitive colonies. Bottom row: samples from the end of a high-density escape time experiment for a vial seeded with only resistant bacteria (left) and a vial seeded with only sensitive bacteria (right). TA, tetrazolium arabinose. (PDF) [file pbio.3000713.s007.pdf]

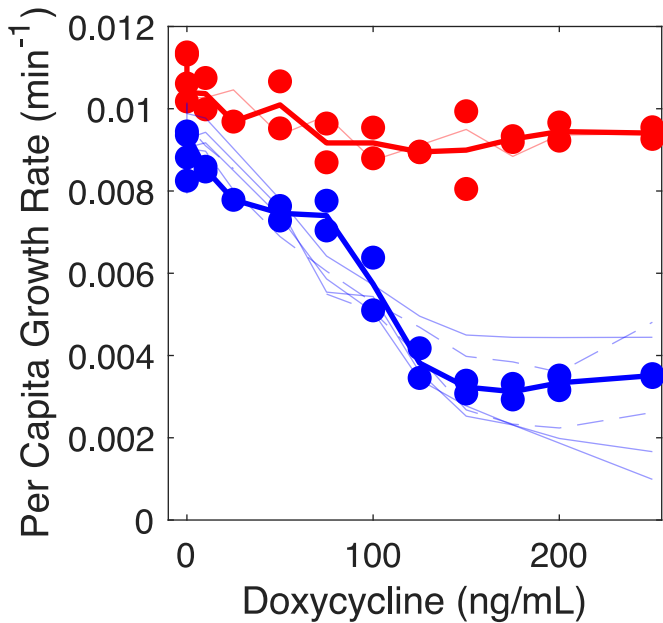

Supplement: S5 Fig — Dose-response curves measured in 96-well microplates for sensitive (REL607; blue circles) and REL606-derived resistant strains (red circles). Dark line, mean across replicates. Thin (transparent) curves correspond to colonies isolated from population mixture at the end of an escape time experiment. Red curve, resistant isolate (appears red on plate); blue curves, sensitive isolates (appear pink on plate). Data are deposited in the Dryad repository: https://doi.org/10.5061/dryad.s4mw6m943 [62]. (PDF) [file pbio.3000713.s008.pdf]

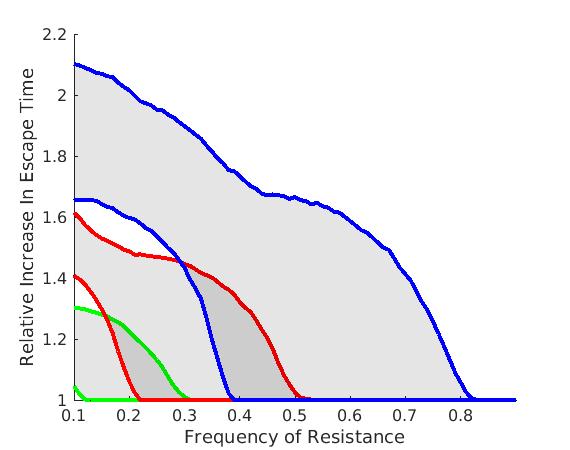

Supplement: S6 Fig — Simulation showing fold increase in escape time gained by using containment instead of elimination. Each shaded region corresponds to a different Pmax (green, red, and blue are 20%, 30%, and 40% of the carrying capacity, respectively). Upper bounds of each shaded region correspond to an intrinsic fitness cost for resistance of 25% (rR = rS(0.75)), and lower bounds assume no fitness cost (rR = rS). Simulation uses mathematical model from main text and parameter values given in Table 1 (except for rR, which is modified as described above). Trends show that increasing the intrinsic fitness cost and decreasing the frequency of resistance will increase the benefit of containment. Importantly, these simulations assume that there is no mutation. The role of fitness costs and frequency of resistance are more complicated when there is appreciable mutational input. Data are deposited in the Dryad repository: https://doi.org/10.5061/dryad.s4mw6m943 [62]. Pmax, acceptable burden. (JPG) [file pbio.3000713.s009.jpg]
