# Supplementary material for: Antibiotics can be used to contain drug-resistant bacteria by maintaining sufficiently large sensitive populations
Source: PLoS Biol. 2020 May 15;18(5):e3000713. doi: 10.1371/journal.pbio.3000713 (PMC7266357; doi:10.1371/journal.pbio.3000713)
Supplement: Attachment — Submitted filename: ReviewerResponseFinal.pdf [file pbio.3000713.s010.pdf]

We thank the editor and the reviewers for their positive evaluations and for their constructive feedback. In response, we have significantly revised the original manuscript which has much improved the paper. Thank you. Most notably, the new edition includes:

1) A great deal of new experimental data testing the containment hypothesis over a range of threshold densities (Pmax). The new data further supports our hypothesis that competition between sensitive and resistant cells can prolong containment times; notably, the quantitative dependence of escape time on threshold density follows the specific predictions of the simple mathematical model (with no free parameters).
2) We include full genome sequencing of the drug resistant isolate as well as the ancestral strain (control) used in this study. We found that the sequence for the control ancestral strain is identical to the published sequence for REL606, as expected. The resistant strain includes five mutations, including an insertion in the transcriptional repressor AcrR which has been previously associated with doxycycline resistance.
3) We've substantially extended our mathematical analysis, providing new insight into the effects of other factors (e.g. fitness cost) on the containment dynamics.

In what follows, we copy the reviewers comments in *italics*. Our responses follow in blue.

We hope you will now find our study suitable for publication in PLOS Biology.

Reviewer #1:

The authors develop a computer controlled bioreactor experimental model to assess whether a strategy of 'containment' (drug treatment only to limit growth below a threshold) can increase time to escape from drug control. They provide exciting experimental support for the containment strategy, illustrating a significant delay to population growth under a containment regime (compared to a limit case of total S annihilation) – even when the containment target is substantially below the bacterial carrying capacity.

These results present an important avenue for future development.

We are happy that the reviewer finds the results to be important.

My two main issues with the current MS are related and fixable primarily in the discussion – first, how could a containment strategy be implemented in the clinic? Second, how does a containment strategy relate to other conditional strategies and to an unconditional low dose strategy?

1) implementation.
The experimental feedback control mechanism doesn't appear to be a plausible clinical strategy (and I'm fine with the proof of concept goals) – but this leaves the key question of translation. What do the authors see as a path to capturing elements of this feedback control in the clinic? It might be interesting at this point to borrow from analogous approaches in the cancer adaptive therapy field, which I believe is more developed. Does the approach work, given uncertainty over the R/S composition of the infection? How can clinicians track infection growth? Imaging? Symptoms??

The reviewer correctly points out that our (primarily proof-of-concept) results raise a number of interesting questions related to potential clinical application. And as the reviewer notes, there are conceptual connections between the current work and adaptive therapy in cancer. In the 5th paragraph of the discussion, we reference an "adaptive strategy" being used in a cancer clinical trial. This adaptive strategy is designed to leverage competition but not necessarily maximize it. Our results (especially the newly added Fig. 5) show that in certain situations competition can very successfully hinder the expansion of resistance, and that increasing competition (for example by increasing Pmax) can improve results. This suggests that, (i) the clinical implementation of

containment does not need to be perfect (simply increasing competition will be beneficial) and (ii) modifying clinical implementations to more closely approximate containment (maximize containment) will improve outcomes. Since clinical implementation of treatment regimens is a complex topic we have not gone into extensive detail about this (our work is a basic science proof-of-concept) but we have modified the 5th paragraph to further clarify these ideas. We have also discussed some of barriers to implementation in lines 328-346.

2) relationship to other conditional/unconditional strategies
A full clinical implementation of this experimental model would require information on the initial resistance distributions and also the temporal dynamics of an infection – this is a tough goal (see point 1). However just having resistance profiles alone could open simple and effective treatments – use a drug that is effective against both co-infecting strains. (https://journals.plos.org/plosbiology/article?id=10.1371/journal.pbio.3000250). In light of this simpler strategy (treat conditional on resistances), why invest in containment? One argument is pan-resistant strains – conditioning on resistance is no longer effective, and conditioning on pathogen growth might buy time for … immune control?

Strategies that capitalize on different mechanisms of drug action and different resistance mechanisms are indeed a critical part of resistance management and control. If a drug combination can be identified that is completely effective then this solves the resistance problem. If, on the other hand, combination therapies are not completely effective then there is still a risk of resistance-induced treatment failure. In that case, it is possible that we could better control resistance by changing how we administer the combination. For example, the drug combination could be administered in high doses in an attempt to lower the pathogen burden as much as possible -- or -- a containment strategy could be used. In this sense the questions we investigate are relevant for combination therapy too. This is an important point and we modified the first paragraph of the introduction to clearly explain the scenarios we are focused on (lines 43-47).

It would also be useful to discuss relationships with the most simple and low tech strategy of reducing drug dose – which will tend to reduce competitive release. This would be an interesting avenue for a control experiment / simulation (same total drug, constant administration)

We simulated the effects of exposing mixed populations to constant drug concentrations and found mixed results (see Figure R1 below). Sometimes these populations "escape" the acceptable burden before the resistant-only vial. Sometimes they take longer to escape than the resistant-only vial but still escape before a containment strategy would. In both of these cases the escaped populations still contain an appreciable number of sensitive cells. Only when drug concentrations were quite high (so high that drug significantly decreases the growth of the resistant strain) did constant drug concentrations control the population for longer than containment would. Note that our implementation of containment did not allow drug concentrations to exceed 125 ng/ml. A constant administration of the highest allowed concentration corresponds to an escape time of about 17 hours (black circle). Which is about 4 hours less than under containment (about 21 hours) – the total amount of drug used under containment is also less. In our experimental model we assumed that drug concentrations could not exceed 125 ng/mL to minimize the effect that drug had on the resistant strain. Capping the maximum drug concentration is also intuitively similar to the idea that drug concentrations in humans cannot exceed a certain maximum value, for safety and to prevent toxicity. For comparison, if we had capped the max drug concentration at 100 ng/ml then the relative benefit of containment is even greater. In this case the max allowed drug concentration fails at about 13 hours compared to 18 hours for containment (see Figure R2).

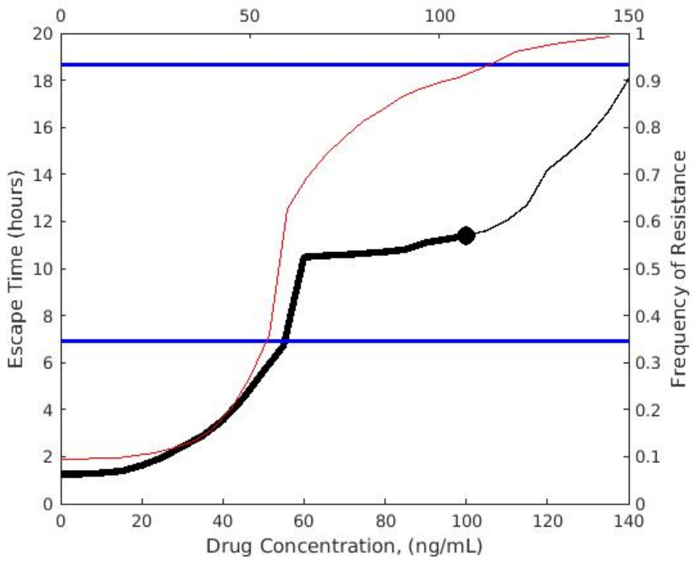

Figure R1 Caption: Escape times for constant drug concentrations. Black curve shows escape time (left axis) for different constant drug concentrations. Simulation is for a high Pmax (50% of carrying capacity) and uses the model and parameters from the manuscript. Time to escape for resistant only vial is about 6.5 hours (indicated by lower blue line) and for the mixed vial under a containment strategy is about 21 hours (indicated by upper blue line). Using a constant drug density can lead to a range of escape times of the mixed-vial: (i) at low concentrations the population escapes sooner than the resistant-only vial (black curve below lower blue line), (ii) for intermediate concentrations the population escapes after the R-only vial but before the containment strategy (black curve between the two blue lines), (iii) for high concentrations the escape time is longer than for containment (black curve above upper blue line). The lower the drug concentration, the lower the frequency of resistance when the population escapes (red curve, right axis) – suggesting treatment failure is not due to resistance. In our experiment, drug concentrations were not allowed to exceed 125 ng/mL (thick black curve indicates allowed concentrations). For the highest allowed drug concentration in our experiment (125ng/mL), maintaining a constant drug concentration results in the population escaping about 4 hours sooner than it would under containment (compare black circle to upper blue line).

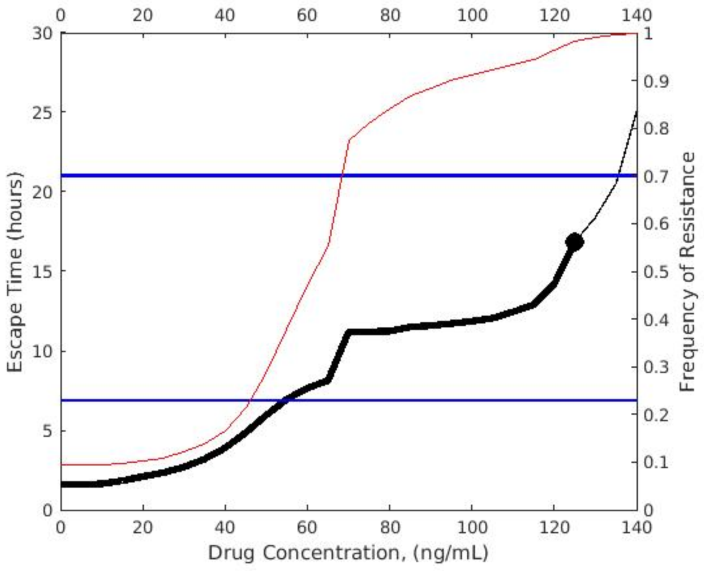

Figure R2 Caption: Escape times for constant drug concentrations. Same as Figure R1 except the max allowed drug concentration for containment is 100 ng/mL. In this case, maintaining the highest allowed drug concentration (100ng/mL) results in the population escaping about 7 hours sooner than it would under containment (compare black circle to upper blue line).

other concerns:

3. *Fig 4: please use time in hours on y axis. The timescale here is important in my view to discuss. You're potentially adding hours to the control window – not a substantial time period. This ties into another key translational theme – what is the clinical advantage of adding hours to the window of time until loss of infection control? In an acute infection context this might be of practical significance for development of adaptive immune control – not sure here, would be interested in authors thoughts.*

The timescale for these experiments is indeed an important factor to discuss. Our experimental results are intended to establish a proof-of -principle and not to quantify the size of effect that would be expected in the clinic; as a result, we caution against drawing specific clinical conclusions from these timescales, which are determined by (for example) the growth conditions in the laboratory bioreactors. Nevertheless, the relative change in escape times can be significant--a factor of 3 or more—depending on the threshold Pmax (see new Figure 5). This suggests that there may be patient-pathogen-drug combinations where the benefit of containment

could be significant--perhaps as you suggest, as a way to buy enough time that the immune system could eventually manage and perhaps even clear the infection on its own. In other cases, even with a large benefit, treatment failure will eventually occur. Sometimes delaying treatment failure when be beneficial for the patient and sometimes – even prolonging treatment duration by 2-3 fold – will be insufficient to make a practical difference. We have added text to discuss this in the introduction (Lines 56-59) and the discussion (Lines 332-333). When discussing Figure 4 we have ben more specific about quantifying the differences in escape time (Lines 230-234).

4) Related to timescale issue - what were the obstacles to running the experiment for longer? It would have been useful to compare the ultimate escape outcomes in all treatments and replicates. More data on longer timecourses would be helpful- the authors should at least comment on what the road block was to longer implementation of this device.

There are some technical difficulties (related to reservoir sizes and risks of contamination) in running experiments for longer than a day. We have now included an example experiment (Figure 5c) where containment was maintained for more than 35 hours. We now discuss this in the Results (lines 247-256) and the Discussion (lines 277-281).

Minor:
5) How resistant is the 'R' E. coli strain? Sounds like a low ½ concentration. Not a big concern as this is plainly a 'proof of concept' implementation.

Figure 2 in the manuscript shows that the resistant strain is essentially unaffected by drug concentrations up to the allowed maximum (125 ng/ml). The IC50 in the mutant strain is increased by approximately a factor of 4.

Reviewer #2:

In the light of the worldwide spread of multi-drug resistant bacterial pathogens, it is important to develop new approaches that limit the spread of superbugs and make current antibiotic therapies more sustainable. The paper by Hansen et al. contributes to this aim and asks whether less aggressive antibiotic treatments, allowing competition between susceptible and resistant strains could repress overall population growth, thereby keeping pathogen burden below an acceptable level and extend treatment efficacy.

I concur with the authors that inter-strain competition could be a powerful way to limit the spread of antibiotic resistant strains. But unfortunately the experimental design and the data presented are quite weak, and I am not convinced that this study will extend our knowledge on the role of competition in making antibiotic therapies more sustainable.

As described below, we have significantly added to the original experimental dataset—an effort that represents nearly six months of additional experimental work—and we also now include genome sequencing to characterize the model strains used in this study. We believe the new results, which extend the previous analysis to a wide range of threshold densities (Pmax), substantially enhance the support for our hypotheses while also illustrating the limitations of the containment approach when threshold density (Pmax) is small. In addition to new experimental data, we have expanded our discussion of both the goals and limitations of the current proof-of-principle study, which we hope will assuage the reviewers concerns about both the experimental design and the ultimate scope of the work.

The authors performed bioreactor (chemostat) experiments with Escherichia coli bacteria. They have three treatments: (1) a monoculture of a susceptible strain, (2) a monoculture of an antibiotic resistant strain, and (3) a mixture of the two. Through the automated adjustment of the flow in the bioreactor, population growth rate can be kept constant under a range of antibiotic concentrations. The authors chose two arbitrary "acceptable" pathogen levels (OD = 0.225 and OD = 0.125), and measured the time for the treatments needed to surpass this threshold. The time needed to do so is called "escape time" and is interpreted as time until treatment failure. The authors found that (i) the escape time was very short for the antibiotic resistant strain, (ii) sensitive populations declined under treatment and never surpassed the threshold, and (iii) mixed populations had either an extended escape time or did not surpass the threshold. The authors conclude that competition helps to keep bacterial load at a manageable level. While I have no problem with these results, I question the overall relevance of the finding.

(1) The authors consider treatment 2 (resistant bacteria only) as a "mutation minimization" treatment, matching aggressive antibiotic treatment. However, aggressive treatment is thought to minimize the risk of resistance to arise in susceptible populations and not in already resistant populations as implemented by the authors. In this context, it is not surprising that resistant monocultures grow best. Anything else would be surprising, and this treatment does not match a "mutation minimization" treatment as one would deploy in clinical settings.

Thank you for this comment. Indeed we should clarify both the goal of the experiment as well as the potential connection to "mutation minimization" treatment. First, the goal: the experiment was designed to directly test whether mixed populations containing sensitive cells can be contained for longer periods than matched R-only populations with identical (starting) numbers of R cells. Our hypothesis was that the presence of the drug-sensitive population may lead to longer containment times because of inter-strain competition; in short, the drug is used to contain the sensitive population, and the sensitive population helps contain the R cells.

The resistant-only vial shows the growth of the resistant population in the absence of sensitive cells. To allow for a direct comparison of containment in R-only and mixed populations, we designed the experiment to ensure that the only difference between the R-only and mixed vials was the absence/presence of sensitive cells. To do this we matched the flows and drug concentrations in all of the vials. This way we could ensure that any differences in escape time are attributed to the effect of sensitive cells. (The drug **may** directly slow growth of resistant cells in the mixed vial, but if this occurs it will also occur in the resistant-only vial because the drug concentrations are the same in all the vials.) Additionally, although our resistant strain is not completely unaffected by drug, Fig 2 of the main text shows that the effect is very negligible for concentrations attained in the vial (maximum 125 ng/ml). We have clarified the motivation for our experimental set-up by adding text on L142-143 and L160-161.

As for the second question: how is this experiment (R-only) connected to the mutation-minimization strategy? The experiment (R-only) represents an idealized version of a mutation-minimizing strategy. It does so by completely removing sensitive cells, mimicking very aggressive treatment (and we term this "mutation minimization" because it eliminates the opportunity for mutation of sensitive cells into resistant cells). Since this can be confusing, we have greatly simplified things by removing all discussion of a mutation-minimizing strategy and, instead, emphasized that we measuring escape times in the presence/absence of the maximal sensitive population (these changes are reflected in lines 142-143, 164-167 and 260-262). We have also changed the section heading on Line 136.

(2) The authors consider treatment 3 as a "competition maximization" treatment, and conclude that competition is only efficient in extending escape time at higher population densities. I have multiple problems with this conclusion. (a) Higher bacterial burden is likely bad for the patient and this is not a practicable way to take.

The reviewer makes several important points which we failed to clarify in the original draft. First, there are absolutely situations where it is not reasonable to maintain a pathogen burden of any size in a patient. But our goal was to explore whether—under controlled laboratory settings—there might be situations where maintaining

a competition-inducing pathogen load could potentially prolong containment. Indeed, there are already clinical situations where pathogen burdens are being deliberately left in the patient (for example physicians sometimes withhold antibiotics for certain urinary tract infections and ear infections), but this issue is by no means settled. Treatment strategies that leverage competition are also being tested in cancer clinical trials (ref. 46 from main manuscript). A primary goal of our work is to determine whether such containment strategies might be viable under controlled conditions and, if so, whether the factors influencing that containment can be understood quantitatively (i.e., a proof-of-principle study on the basic science underpinning an area of immense applied importance). The surprising part of our results is that even under well-stirred and spatially homogeneous conditions, the presence of sensitive cells can have a substantial impact on the escape time. What's more, the quantitative dependence of this effect on the burden level (Pmax) follows predictions of a simple competition model and can therefore be predicted once basic growth parameters (e.g. IC50 of the drug in each strain) are measured. We have added an entire section in the Discussion to discuss these issues (lines 328-345).

(b) the chosen thresholds are arbitrary and the growth increase observed for the mixed treatment under low density (Fig. 3C) is minimal. It surpasses the threshold, yes, but compared to the resistant only treatment, mixed cultures are still much more stable and show a dramatically lower growth increase. Thus, competition also has an effect at low cell density.

We have now **included extensive experimental results (Figure 5)** that look at the effect of the sensitive population on the resistant population for a wide range of acceptable burdens (Pmax). This allows us to quantify how the effect of competition changes as you increase the total population size. **We explain this in more detail in our response to your comment (3) below.**

(c) the observed effects are transient. The authors plated the mixed cultures at the end of their experiment and found that populations are dominated by resistant clones. This means that in the long-run competition cannot constrain the spread of resistance.

Yes, the reviewer is correct that in our experimental system the resistant strain usually expands to exceed the acceptable burden. Hence, the expected benefit of containment is transient (mathematically, it increases the escape time but does not alter the steady state fixed point). In a clinical setting these results may or may not be transient and will depend on factors such as fitness costs and immunity. Even if the resistance eventually leads to treatment failure – increasing the time to treatment failure may be beneficial to the patient. We have added more information about this in numerous places in the manuscript (L56-59, L249-256 , L291-296 and L332-333).

(3) The advantage of in vitro proof-of-concept studies is that there is full control over the experimental set up and a large number of conditions and treatment combinations can be tested. Unfortunately, the authors did not make use of this advantage and the data presented is thin both in terms of quality and quantity. (a) The authors use an undefined laboratory evolved antibiotic resistant mutant. We don't know the resistant mechanism involved and the potential mechanisms of competition that could drive interaction patterns in mixed cultures.

We have now characterized the resistant strain and added this information to the manuscript (lines 123-126 and L409-425). Also, the purpose of our experiment is to provide proof of concept that *in the appropriate situations* containment strategies can slow the expansion of a drug resistant population. The particular genetics of the pathogen-drug combination that occurs in our model system is not the objective of the study.

(b) Only one antibiotic was used for hypothesis testing. So we don't learn whether the observed patterns are of general relevance or specific to this antibiotic.

While it was not feasible to repeat the study in multiple antibiotics, we hope the reviewer will recognize that even under in-vitro conditions, these experiments represent a considerable undertaking. We have a grant pending at the NIH to apply our approach to other antibiotics.

(c) Only two populations densities were examined and we thus don't learn what the overall relationship between escape time and population density is. This is particularly problematic because OD = 0.2 is called high density although in absolute terms OD = 0.2 is still very low density. From a proof-of-concept study, which is far away from anything that is clinically relevant, I would expect a much larger parameter space to be explored, especially with the mathematical model at hand.

The new manuscript includes substantial new experimental data (Figure 5 and lines 239-256)—more than doubling the size of the data set and including a wide range of Pmax values. These new experimental results closely match predictions from our competition model and demonstrate the quantitative relationship between containment time and Pmax.

More minor comments:

4)- It is unclear what should be extracted from the left panels of Figure 2. Is this just to demonstrate that real-time growth rates can be measured in the chemostats? This is quite trivial because this is what chemostats are made for.

While we see the reviewer's point, we have found that readers are sometimes confused by the different "chemostat-like" set-ups that can be used to measure growth (e.g. turbidostats, like what we used here, vs chemostats vs morbidostats). We have elected to keep the panels in hopes of preventing any confusion about the growth measurements and to illustrate the connection between the dose response curves and the raw data.

5)- Why does the resistant strain treatment start with a much lower OD than the other two treatments? This induces a massive bias towards longer escape times for this treatment.

The experiment compares escape time between (i) a resistant population and (ii) a resistant population (of the same size) with a sensitive population added. The goal is to test whether the presence of the sensitive population will slow the expansion of the resistant population. Intuition indeed suggests that there is a bias towards larger escape times for the resistant-only vial in the absence of competition or other intercellular interactions. Surprisingly, however, our results show the exact opposite: the escape time for the resistant-only vial is smaller than that for the mixed vial (provided enough sensitives are added…i.e. a sufficiently large Pmax). In this sense, the experimental design is actually biased *against* the results we found, as the advantage (extended escape time) conferred by sensitive cells must overcome the inherent disadvantage that comes from starting with a larger population that is closer, at time 0, to the escape threshold. We have helped to clarify this by adding L142-143.

6)- page 2, line 3: "aggressive treatment may actually promote the emergence of resistance" sounds like the treatment itself will actively give raise to resistance mutation. Consider rephrasing to "promote selection for resistance" or "promote the spread of resistance"

We have made this change.

7)- end of page 2 and beginning of page 3: I don't think that statements like "this is the first explicit demonstration" and "we are also unaware of any direct tests …" really help to increase the impact/relevance of the paper. Please remove.

We have made this change.

8)- page 3: “the celebrated long term evolution experiment” reads odd. Please be more objective.

We have made this change.

Reviewer #3:

The aim of this study is to provide a proof-of-concept experimental illustration that under given conditions, containment (i.e. more moderate) drug regimens can better manage antibiotic resistance than elimination (i.e. aggressive) strategies. For this the authors design three scenarios in an in-vitro experimental setup with automated drug and nutrient delivery to study the effect of treatment strategy on resistance emergence: i) resistant-only (no competition), ii) resistant+sensitive cells (competition scenario) and iii) sensitive-only dynamics. They set the drug concentration adaptively in the mixed scenario, to maintain the population at a certain density, and apply the same drug concentration to the other homogeneous populations. The readout for time of escape is the time it takes each population to cross a given target threshold density. The study finds that in the mixed case, the time of escape is higher than in the resistant only case, thus indicating the important role of competition in delaying resistance selection in real settings. This finding is also corroborated by a mathematical model. The authors go on to test this effect at another population size, and report that at lower population sizes the benefit of competition in reducing resistance is lower. Overall, I consider the study interesting and novel in the combination of ecological theory, experimental in vitro-approach with computer-controlled bioreactors and mathematical modeling. Indeed, the question of optimal drug therapies is a very urgent one in the face of antibiotic resistance. Previous mathematical models using population dynamic arguments have shown that competition between drug-sensitive and drug-resistant pathogens can slow down the selection of resistance. This particular study goes one step further by providing experimental validation of this expectation. However, there are some parts of the study and analysis that I find confusing and that I would need the authors to clarify.

1. I am a bit confused by the authors' focus and repeated reference to "emergence of resistance" and "escape time", since they do not talk explicitly about the mechanisms underlying this escape. Is it because of selection of pre-existing sub-populations, or because of further or de-novo mutations? In particular, are the mechanisms of "escape" the same in the Resistant-only, mixed case, and Sensitive-only scenarios? In general I guess not, and I would invite the authors to comment a bit more on this biological aspect. If I understand it correctly, in the Resistant-only scenario, the process governing escape is basically growth of resistant cells under sub-inhibitory antibiotics and the time it takes to exceed a certain population size threshold. In the mixed case, escape is basically due to the selection of the resistant sub-population in the context of a heterogeneous population, where the majority of sensitive cells by competitive suppression, delay any growth advantage of the resistant minority in the presence of the drug. In the sensitive-only case, escape can only happen if there is an allegedly very rare beneficial mutation conferring drug resistance in the time frame of the experiment followed by subsequent growth. Thus, there should be no emergence per se in this study, only selection. This is also confirmed by the dose-response curve for R-only population at the end of the experiment, which is the same as the original strain.

Your assessment is completely correct. We were using ``emergence" to mean ``expand to exceed the acceptable burden". We admit that this is not a standard usage of the term ``emergence" and can be confusing. Thank you for pointing this out. To improve clarity we now use terms like ``expansion of the resistant population"; these changes are reflected in lines 50, 73, 87 and 271.

2. Another issue the authors seem to overlook is the fact that mutational processes can happen both in the sensitive cells and already resistant cells. There are many studies that show that drug-resistant cells may experience even higher mutation rates that effectively accelerate further resistance evolution. In the present study, this process is completely neglected. Why?

We haven't explicitly discussed these ideas because we are focused on the situation where the resistance presence is already high enough that drugs are not effective. (We have helped to replicate this scenario by capping the maximum drug concentration in our vials to 125 ng/mL – Fig 2 from the main text shows that drug concentrations at this level have minimal effect on the resistant strain). If levels of resistance are only intermediate, then drug can be used to suppress expansion (we would classify these cells as part of the sensitive population, and they would modify the average properties of the sensitive population). Lines 43-47 now help clarify the focus of our study. We have also removed references to "mutation minimization" (see our response to comment 1 from Reviewer 2.

Additionally, the characteristics of our resistance population does not seem to change much during the experiment (we have added lines to highlight this L274-281).

3. Regarding, the definition and approach to competition adopted in this study, I would also invite the authors to elaborate more and be more explicit on:

i) the confounding between population size and heterogeneity in their 3 scenarios: Besides the fact that we are dealing with a homogeneous vs. heterogeneous population, there is also a big difference in initial population size in the R-only (much lower than Pmax) and mixed case (equal to Pmax). This by default creates an advantage for the population of cells growing in the R-only regime. And this advantage is higher, the more space there is for growth from P(0) to Pmax. Thus, in one case we are dealing with a growing ('epidemic scenario') population, in the other case, we are dealing with an 'endemic' scenario, of constant population size and simple replacement. It is known also from epidemiological models and studies that patterns of evolution can be different.

Please see our response to comment 5 from reviewer 2.

ii) What is the rationale behind equalizing the drug concentrations across the scenarios? By equalizing drug concentrations (set by the mixed regime) the authors are comparing dynamics using minimal inhibitory treatment vs. sub-inhibitory treatment relatively in the "mixed/sensitive-only" and R-only scenarios. Thus, unlike the schematic shown in Figure 1A, the resistant-only scenario, is experiencing much lower concentration of drug than its corresponding aggressive regime, amplifying the total growth advantage relative to the mixed case. Thus, not only they are starting with $dR/dt>0$, not only resistant cells are not competing with sensitive cells, they are also under less drug-inhibition overall. It seems that the authors are simulating in the R-only regime a treatment of the type "assumed prior aggressive treatment that removed the S population + moderate treatment when there is only the resistant population left".

Please see our response to comment 1 and comment 5 from reviewer 2.

iii) Competition maximization to slow down resistance is one thing, infection clearance is another. What are the novel prospects for infection clearance generated by this study? In light of this work, could addition of drug-sensitive cells to a drug-resistant infection be used to increase competition and leverage its benefits?

Infection clearance may be possible in certain situations (for example if immunity is eventually able to control the infection). We have added text to highlight this (lines 56-59 and L332-333).

Theoretically, adding drug-sensitive cells to a drug-resistant infection be used to increase competition. Actual examples that come close to this idea even exist. For example, fecal transplants. There has also been some investigation of using non-virulent *E.coli* to help manage recurrent UTI infections. We haven't explicitly stated this idea in the manuscript, because simply treating less and deliberately maintaining a burden is still contentious (see for example comment (2) from reviewer 2). Instead of discussing **deliberately adding** sensitive cells to an infection, we have added a section in the Discussion which discusses the idea of **deliberately** leaving sensitive cells in an infection (L 328-345)

4. It is necessary for this study to address more clearly what the effect of containment strategies is along the 2-d continuum: pop. size vs. initial frequency of R. The authors explore indeed two population sizes (achieved with different drug dosing), but these correspond at the same time to two different initial frequencies of resistance in the bacterial population, namely 10% in the high population size and 20% in the small population size. Thus, we cannot really disentangle whether the sensitivity of escape time is a population size effect or frequency-dependent effect, or of both. The higher the initial frequency of resistance in the mixed case (i.e. here the lower the Pmax), the closer we are to the R-only extreme, and that is why no difference is found in "escape time." I recommend the authors to comment on this issue when interpreting their results.
In my view, a contour plot of the model-derived expected time to escape as a function of both population size and initial frequency would be an important and insightful addition to the paper. I would even suggest to provide two versions of such contour plot, for example by changing the growth differential between the sensitive and resistant strains or efficacy of antibiotic. In this way, it will become clear that the quantitative benefit of containment does not depend just on population size (as suggested in the paper and abstract), but is indeed much more complex, and may vary also with all these other parameters and initial conditions.

Additional factors such as the amount of resistance and fitness costs will definitely impact the effect that sensitive cells has on the expansion of the resistant population. We have previously mentioned the importance of the amount of resistance in the Introduction L76-85.

We have now also added an additional section to the Discussion (L282-296). We also include (in this response) a simulation to demonstrate the effect of fitness costs. We are hoping to investigate these and other related effects experimentally in future work, though it is a considerable undertaking.

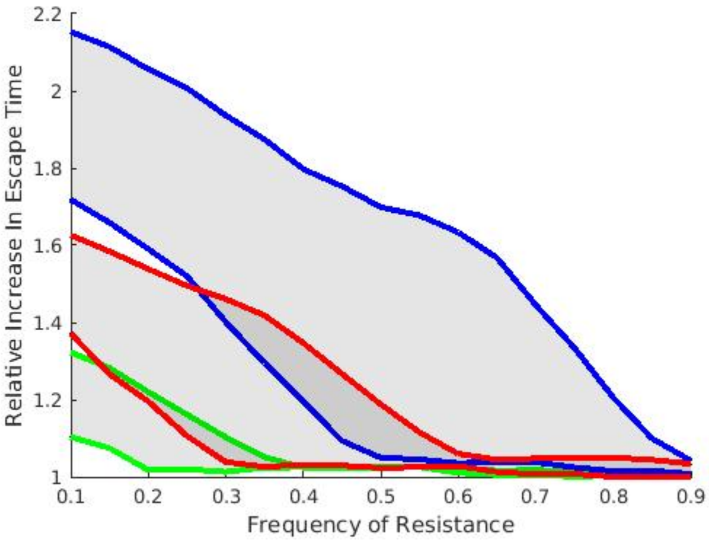

Figure Caption: Amount of benefit from containment depends on multiple factors. Y-axis shows the fold increase in escape time gained by using containment instead of elimination. Each shaded region corresponds to a different Pmax (bounded by green: Pmax is 20% of carrying capacity, bounded by red: Pmax is 30% of carrying capacity,bounded by blue: Pmax is 40% of carrying capacity,). Upper bounds correspond to an intrinsic fitness cost for resistance of 25% (r_R=r_S(0.75)), lower bounds assume no fitness cost (r_R=r_S). Simulation using model described in main text and parameter values given in Table 1 (except for r_R which is modified as described above). Trends show that increasing the intrinsic fitness cost and decreasing the frequency of resistance will increase the benefit of containment. Importantly, these simulations assume that there is no mutation. The role of fitness costs and frequency of resistance are more complicated when there is appreciable mutational input.

5. The worst-case scenario for containment is when the total bacterial population is resistant (frequency of resistance=1) and when the population size is large. This scenario cannot be tested with the current framework because the maximum dose feasible is well below the half-maximum inhibitory concentration for the resistant population (129<209). Can the authors comment on this scenario? What would be the role of competition in that case?
Since this study assumes that sensitive and resistant cells compete equally, can we expect a positive effect of competition also in the R-only adaptive regime? Would a moderate regime delay further resistance evolution compared to an aggressive regime?

As mentioned in our response to this reviewer's comment (2), we are focused on the case where resistance cannot be controlled with drug (please see our response there for further explanation).

6. Some more links with existing models from the literature are warranted, e.g. Colijn and Cohen (eLife 2015, https://doi.org/10.7554/eLife.10559.001) where competition between a drug-sensitive strain and drug-resistant strain is modeled in more detail. Similarly, Gjini & Brito (Plos Comp. Biol. 2016 https://doi.org/10.1371/journal.pcbi.1004857) also consider in detail adaptive treatment, and along the lines of this study, conclude that at higher symptom thresholds, where indirect competition via immune stimulation is maximized, resistance selection risk is also lower, as opposed to lower symptom thresholds. This may be relevant for in vivo settings where antigen-dependent immunity acts as another layer of competition on heterogeneous bacterial populations.

Thank you for the references; we have added them. We do see our empirical work as a significant addition to the hitherto wholly theoretical literature. We have also explicitly mentioned the important role of immunity in two places (L56-59 and L332-333).

Similarly, some discussion on the possibility of asymmetric competition is also needed. Here it is assumed that the carrying capacities are the same for both S and R. Indeed, if the competition were asymmetric and S could inhibit S more or less than inhibiting R, and so on (there are in principle 4 pairwise competitive interactions), additional indirect effects could manifest on R dynamics due to S removal by the drug. This phenomenon has been discussed widely in pneumococcus epidemiology and serotype replacement due to vaccination, where asymmetric competition can accelerate or slow down non-vaccine type 'selection' (similar to R selection in the mixed experimental setting of this study).

We agree that differences in competitive ability are important. We have now mentioned that differences in competitive ability can affect results (L282-296). We have not gone into extensive detail about this because (in general) exactly how these differences play out will depend on how they impact the balance of mutational input and competitive suppression. (Differences in competitive ability can impact the amount of mutational input because mutational input can be dependent on sensitive replication.) Since our mutational input is negligible in our model system, detailed discussion of this topic is better addressed elsewhere.

Minor comments:

- Why is the main result in Figure 4 not divided in Probability of Escape and Time of Escape | Escape?

We think this comment is probably related to the non-standard way that we used the term "emergence". Our resistant populations never go extinct and so escape will always occur unless the steady state of the system is below Pmax. The resistant populations shown in Figure 4 always expand to exceed Pmax (i.e., have a well defined escape time).

- In Table 2, the R-only and S-only initial conditions have to be corrected. Please also put the initial conditions in terms of bacterial concentrations as required by the differential equation model.

This change has been made.

- More details on the Model fitting and parameter estimation, especially the delays associated with drug effect on resistant strain and sensitive strain are needed. How important are the assumed differences in these delays?

The differences in the delays are not important. Our fitting algorithm produced different delays for the different strains, so these are the ones that we used. Below is a figure showing the model curves using the parameters in the manuscript as well as analogous curves assuming both strains have the "resistant strain's" delay (red curves) or the "sensitive strain's delay" (blue curve).

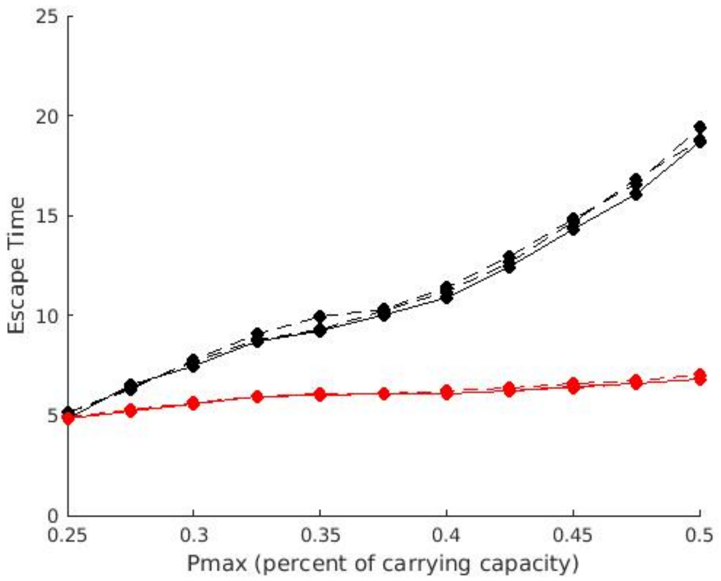

-Figure 3 should be accompanied in the Supplements with the corresponding underlying dynamics of replacement of S by R in the mixed regime during drug administration. The R-dynamics in that case could be compared with the R-only dynamics by overlaying the two graphs.

We have now added this in the new Figure S4.

- In Figure 3, please clarify what generates the variability in model predictions. What is the stochastic component of the simulations?

Each individual experiment includes a control vial with no drug and no inflow or outflow. This control allows us to estimate the carrying capacity. Because of day-day fluctuations in uncontrollable variables (like temperature) the carrying capacity can be different for different experiments (ranges between optical densities of 0.3 and 0.4). The change in the estimated carrying capacity is what produces the different results in the mathematical model. We have updated our parameter table to accurately reflect this. Thank you for noticing this.
