# Supplementary material for: Antibiotics can be used to contain drug-resistant bacteria by maintaining sufficiently large sensitive populations
Source: PLoS Biol. 2020 May 15;18(5):e3000713. doi: 10.1371/journal.pbio.3000713 (PMC7266357; doi:10.1371/journal.pbio.3000713)
Supplement: Attachment — Submitted filename: ReviewerResponseRd2.pdf [file pbio.3000713.s011.pdf]

We would again like to thank the editor and the reviewers for their helpful comments. We have incorporated all suggested changes in the revised manuscript. We hope you will now find the paper suitable for publication in PLOS Biology.

In what follows, *the reviewers comments are in italics* and our responses are in red.

Data:
We have deposited source data for figures at Data Dryad, and we have referenced this dataset in all figure captions. The link is currently private (until acceptance), but the temporary link is below.

Full citation:
Hansen, Elsa et al. (2020), Antibiotics can be used to contain drug-resistant bacteria by maintaining sufficiently large sensitive populations, v2, Dryad, Dataset, https://doi.org/10.5061/dryad.s4mw6m943

Temporary Link:
https://datadryad.org/stash/share/kE43w4pCylBQSq3XOR0fkc56tu1ALCYonMzcnS5wSls

Reviewer #1:

The authors have made valuable edits in response to my earlier comments. I have no further concerns.

minor: fix typo in title of figure 5 (which is a very nice addition)

We have corrected the typo in Figure 5. We thank the reviewer for helping us improve the manuscript.

Reviewer #3:

I appreciate the effort gone into this revision and the clarifications made by the authors to address most of the points raised. I do believe this has improved substantially the manuscript. While still some caveats remain, the authors have mentioned them more transparently in this version, opening the space for future work to fill the gaps. Overall, this is a valuable contribution to the empirical understanding of competition and intervention processes on the dynamics of bacterial populations, which may help address therapeutic challenges for resistant infections in clinical settings.

We thank the reviewer again for helping us improve the manuscript.

- As a further clarification on Figure 3, the provided Figure S4 is not what I had in mind when I asked for a comparison between Resistant dynamics visualization in the R-only vs mixed case. In its current version, I don't find figure S4 informative. I understand the explicit R-dynamics in the mixed case may be inaccessible in retrospect, thus making my request unfeasible with the current data, where only total population size is tracked over time. I would recommend to remove figure S4.

We have removed figure S4.

- I would appreciate seeing the figure about Frequency of Resistance and escape time in the response file (response to my previous comment 4) as an added Figure to the Supplements, with appropriate referencing in the main text.

We have now added the suggested figure in the SI (S6 Fig).

- I suggest removing unnecessary brackets from the model equations (e.g. from the numerator in some fractions), this would make the reading of the model less cumbersome.

We have made these changes.
